# Right thyroid hemiagenesis with adenoma and hyperplasia of parathyroid glands -case report

**DOI:** 10.1186/1472-6823-12-29

**Published:** 2012-11-13

**Authors:** Merima Oruci, Yasuhiro Ito, Marko Buta, Ziv Radisavljevic, Gordana Pupic, Igor Djurisic, Radan Dzodic

**Affiliations:** 1 Surgical Oncology clinic, Institute for Oncology and Radiology of Serbia, Pasterova 14, Belgrade, 11000, Serbia; 2 Department of Surgery, Kuma Hospital, 8-2-35, Shimoyamate-dori, Chuo-ku, Kobe, 650-0011, Japan; 3 Department of Clinical Research, Brigham and Women’s Hospital, Harvard Medical School, Boston, MA, USA; 4 Department of Pathology, Institute for Oncology and Radiology of Serbia, Pasterova 14, Belgrade, 11000, Serbia; 5 University of Belgrade School of Medicine, Belgrade, 11000, Serbia; 6 Institute for Oncology and Radiology of Serbia, Pasterova 14, Belgrade, 11000, Serbia

**Keywords:** Right thyroid hemiagenesis, Parathyroid adenoma, Parathyroid hyperplasia, Hyperparathyroidism

## Abstract

**Background:**

Thyroid hemiagenesis is a rare anomaly, more commonly seen on the left side (ratio 4:1) and in females (ratio 3:1). The first to describe this anomaly was Handfield Jones in 1852.

**Case presentation:**

We present a 66 year old female patient with right thyroid hemiagenesis, parathyroid adenoma on the side of hemiagenesis and parathyroid hyperplasia on the contralateral side. The patient had neck pain and was diagnosed as Hashimto thyroiditis with hyperparathyroidism. Parathyroid hormone, thyroglobulin antibodies (Tg-Ab) and thyroid peroxidase antibodies (TPO-Ab) were elevated. Neck ultrasound and technetium 99mTc-methoxyisobutyl isonitrile (MIBI) scintigraphy confirmed the right thyroid hemiagenesis, but not adenoma of parathyroid glands. Intraoperatively, right thyroid hemiagenesis was confirmed and left loboistmectomy was performed with removal of left inferior hyperplastic parathyroid gland. Postoperative PTH (parathyroid hormone) levels were within normal range. Five months after the operation PTH level was elevated again with calcium values at the upper limit. MIBI scintigraphy was performed again which showed increased accumulation of MIBI in the projection of the right parathyroid gland. Surgical reexploration of the neck and excision of the right upper parathyroid adenoma was performed which was located behind cricoid laryngeal cartilage. After surgery a normalization of calcium and PTH occured.

**Conclusion:**

From available literature we have not found the case that described parathyroid adenoma on the side of thyroid hemiagenesis,with parathyroid hyperplasia on the contralateral side.

## Background

Thyroid hemiagenesis is a rare anomaly, more commonly seen on the left side (ratio 4:1) and in females (ratio 3:1) [1]. The true incidence of hemiagenesis is not known because it is usually asymptomatic and it is incidentaly revealed due to certain pathologic conditions of the contralateral lobe. The prevalance of thyroid hemiagenesis in the literature varies between 0,2% to 0,025% [2-5]. The largest series of 40 thyroid hemiagenesis was published by Ruchala et al. [6]. The same author also performed a study showing that thyroid hemiagenesia was associated with slightly enhanced C cells hyperplasia compared to controls, which might indicate compensatory proliferation, however, the calcium-phosphate balance did not seem to be significantly affected [7].

Thyroid hemiagenesis anomaly was described for the first time in Europe 1852 by Handfield-Jones [8] and later in U.S.A. by Marshall in 1895 [9]. The absence of one thyroid lobe is usually asymptomatic and is often being diagnosed incidentally or during assessment for thyroid related or non-related conditions.

Maganini and Narendran were the first to decribe in the year 1977. case of upper left adenoma of the parathyroid gland in a patient with left thyroid hemiagenesia [10]. Teresa Kroeker published the case report of left lobe hemiagenesia and ipsilateral parathyroid adenoma [11]. Mydlarz et al. published in 2010.case report of ipsilateral doouble parathyroid adenoma and left thyoroid hemiagenesia [12]. The case report of parathyroid adenoma on the contralateral side of hemiagenesis was published by Sakurai et al. And they described the absence of parathyroid glands on the side of hemiagenesis [13]. Duh et al. described thyroid hemiagenesis, together with parathyroid hyperplasia [14].

We report a case of parathyroid hyperplasia and adenoma, hyperparathyroidism, Hashimoto thyroiditis, and rare right thyroid hemiagenesis.

## Case presentation

**Figure 1:**
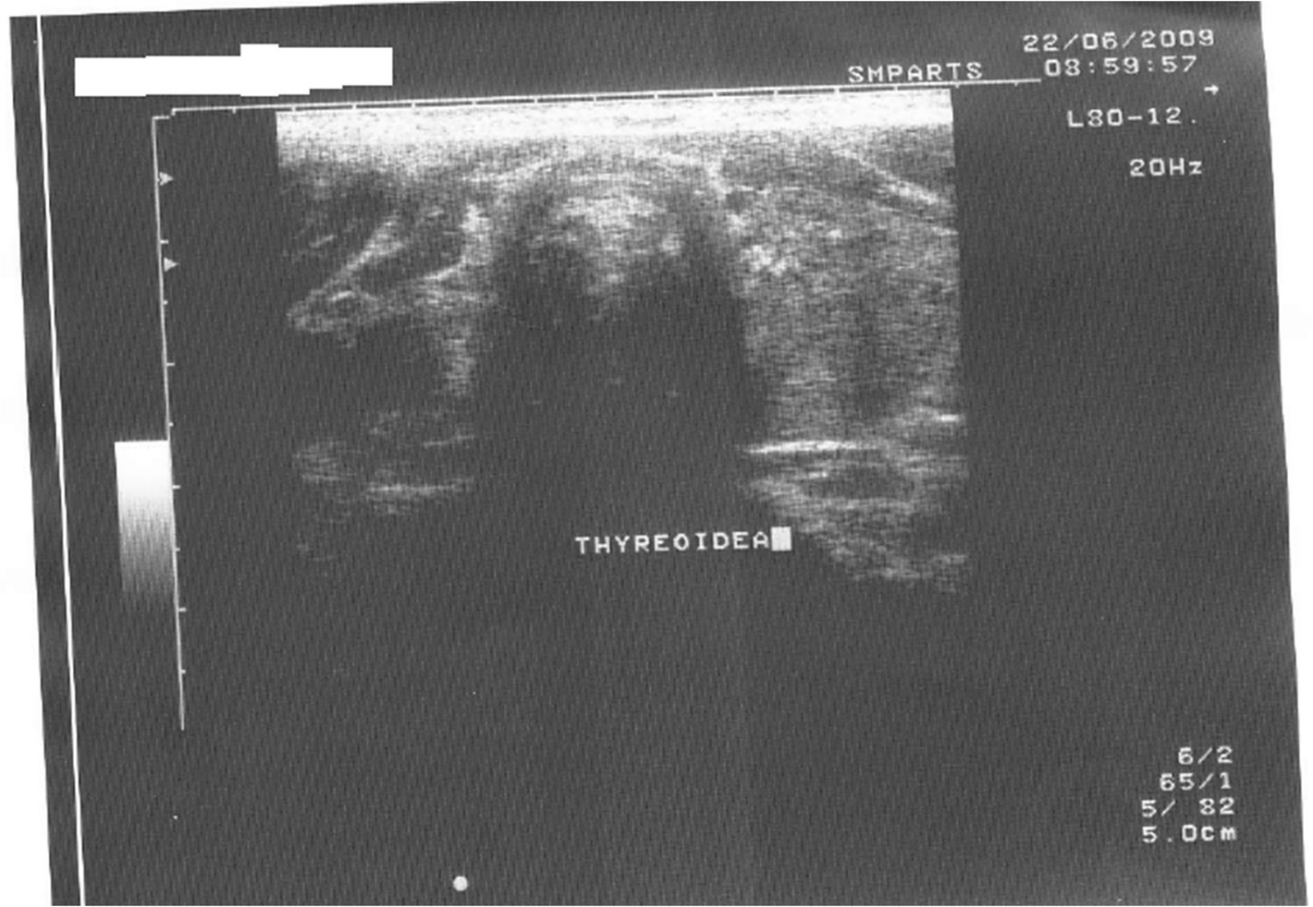
Neck ultrasound.

**Figure 2:**
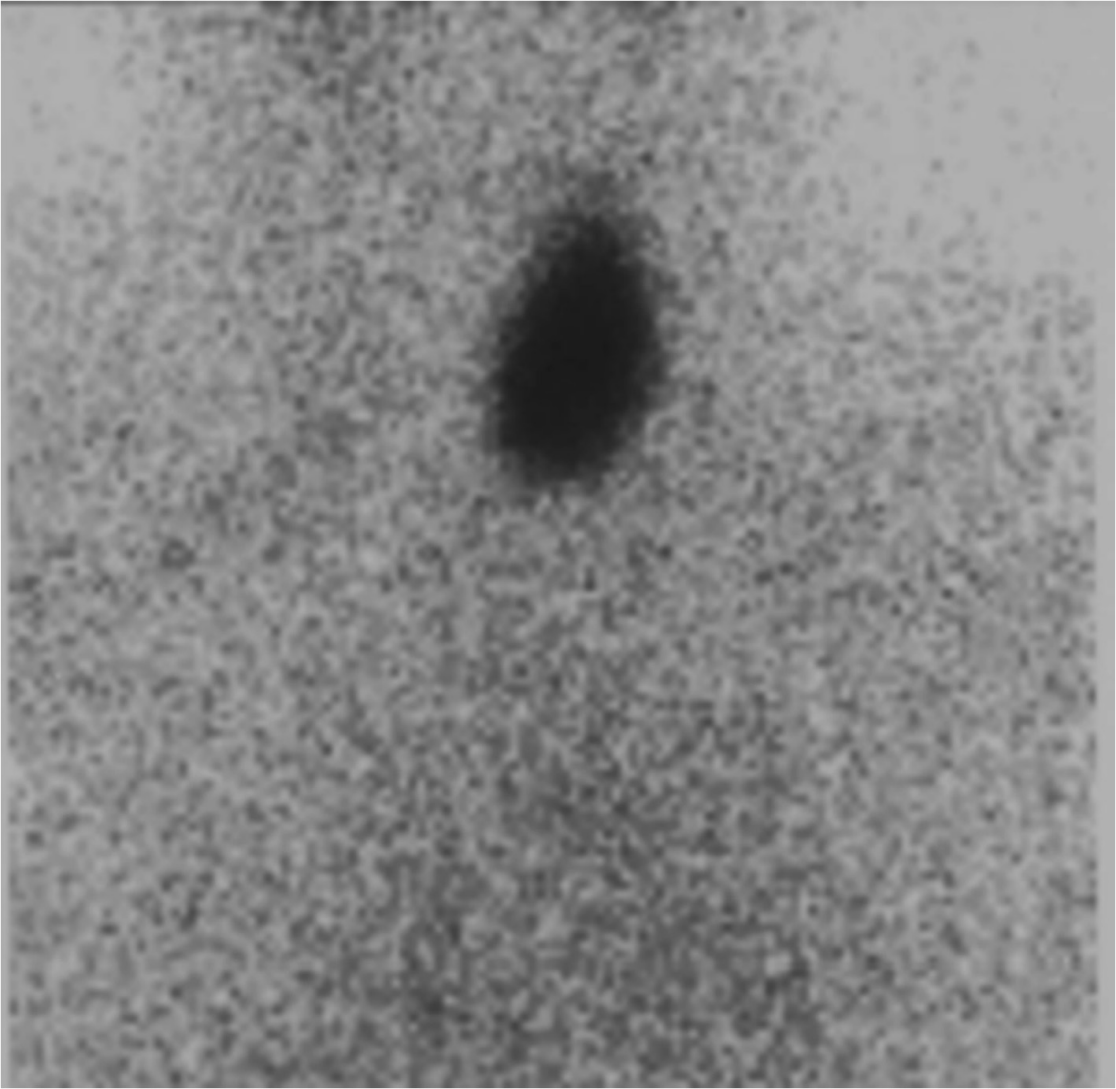
Neck scintigraphy (MIBI).

**Figure 3:**
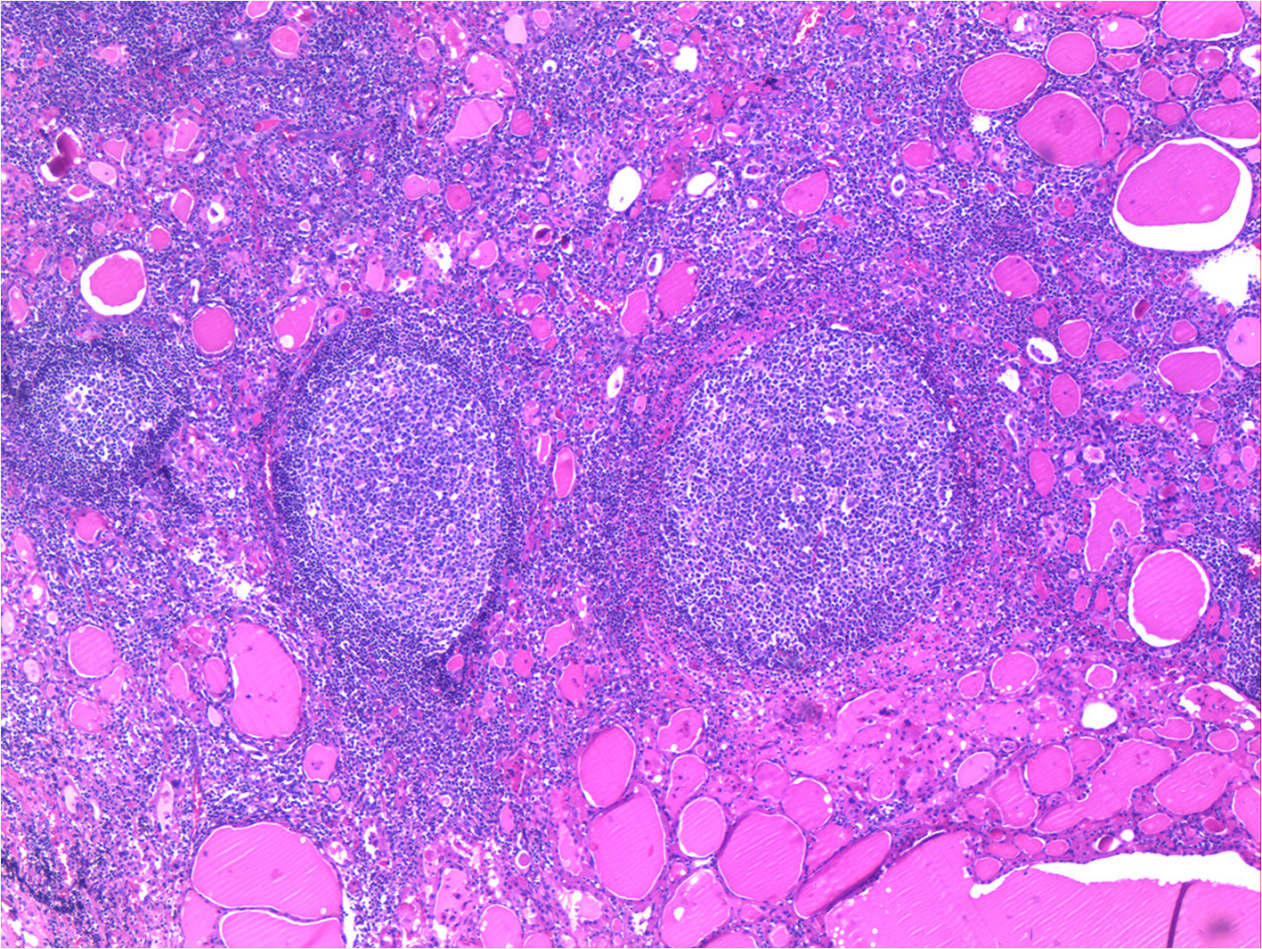
Hashimoto thyroiditis HE-staining, 20× magnification.

**Figure 4:**
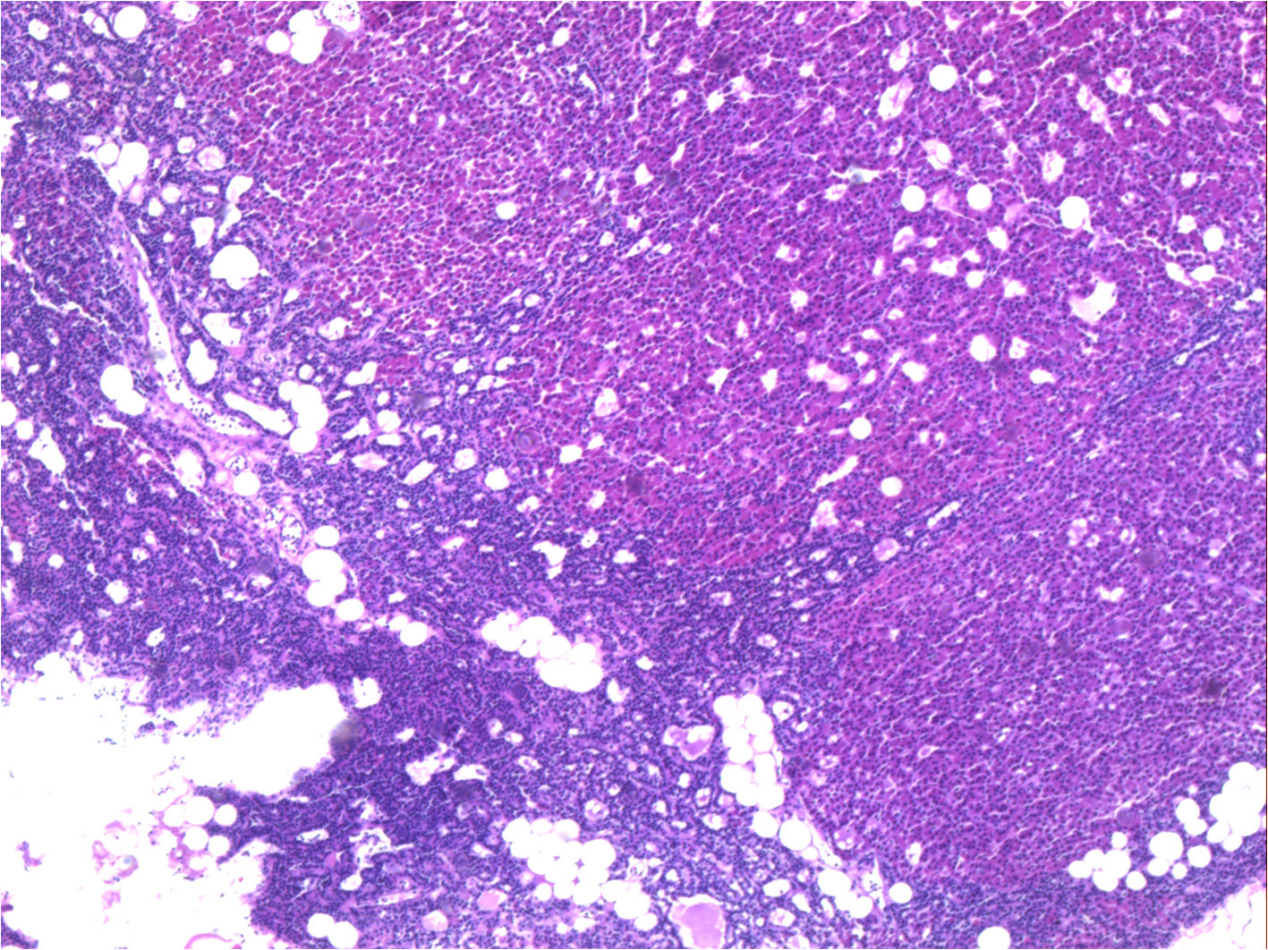
Hyperplasia of parathyroid gland HE-staining, 20× magnification.

**Figure 5:**
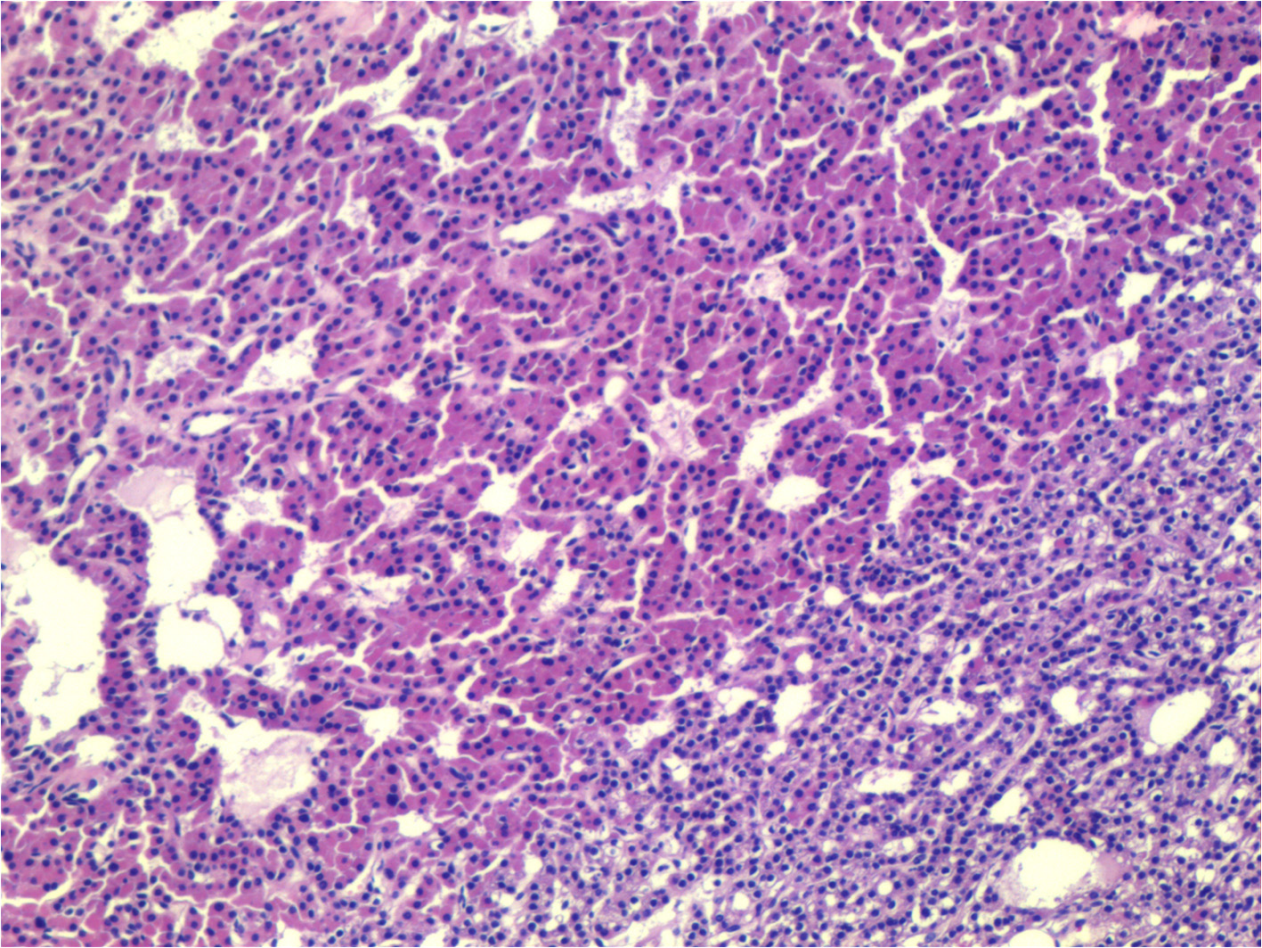
Adenoma of paratyroid gland HE-staining, 20× magnification.

A 66-year-old woman was diagnosed with primary hyperparathyroidism, Hashimoto thyroiditis, and tumor in the left thyroid lobe in July 2009. There was no family history of thyroid and parathyroid disease. The parathyroid hormone (PTH) was elevated (136.2 pg/ml vs. normal value of 15–65 pg/ml) as well as calcium (Ca) level (2.73 mmol/L vs.normal value of 2.15-2.55 mmol/L). Also, thyroglobulin antibodes (TG-Ab), thyroid peroxidase antibodies (TPO-Ab) and thyroid stimulating hormone (TSH) (17.58 microU/ml vs. normal value of 0.27-4.2) were elevated, but L-thyroxine (T4) level was decreased (64.89 nmol/L vs. normal of 66–181 nmol/L). The patient was treated by L-thyroxine50 μg daily. The patient did not have nephrolithiasis or osteoporosis. Ultrasound of the neck verified absence of right thyroid lobe with heterogeneous structure size of 23x45 mm in the left lobe and enlarged lower left parathyroid gland size of 8x6 mm (Figure 1). Fine needle aspiration biopsy (FNAb) was not performed and the decision for the operation has been made only based on clinical and ultrasonographic findings. Technetium 99mTc-methoxyisobutyl isonitrile (MIBI) scintigraphy of parathyroid glands initially showed no pathological accumulation and only the left thyroid lobe could be visualized. (Figure 2). Tc99 was injected at the day of surgery. A left thyroid lobectomy and left lower parathyroidectomy were performed, both showing increased Tc99 accumulation. Exploratory surgery confirmed agenesis of the right thyroid lobe. Histopathologic examination confirmed Hashimoto thyroiditis of left lobe (Figure 3) and hyperplasia of the lower left parathyroid gland (Figure 4). Postoperative levels of calcium, PTH and phosphorus were normal. Five months later PTH level was increased again to 145 pg/mL. MIBI scintigraphy of parathyroid glands was performed again and pathologic accumulation was seen in the right parathyroid gland. Patient was reoperated and adenoma of the upper right parathyroid gland was removed size of 15x8 mm, located behind the crycoid cartilage (Figure 5). The right lower parathyroid gland was found and was normal in size and structure. Postoperative PTH, serum Ca and phosphorus (P) levels were normalized and their values still remain within normal range two years after the surgery.

## Discussion

Here we report a rare case of right thyroid hemiagenesis, Hashimoto thyroiditis, hyperparathyroidism due to parathyroid hyperplasia and adenoma. Thyroid hemiagenesis was described previously with hyperparathyroidism [14], Hashimoto thyroiditis [15], thyroglossal duct cyst [16], follicular and papillary neoplasms [17,18]. Diagnosis of thyroid hemiagenesis can be easily accomplished by Tc-99m MIBI scintigraphy and ultrasonographic examination. In our case Tc-99m MIBI scintigraphy did not detect parathyroid adenoma before the left thyroid lobe was removed and we can speculate that the left lobe absorbed all radioactivity. Only after totalisation of thyroidectomy by removing the left lobe, right parathyroid adenoma could be seen. Explorative surgery was necessary for the final diagnosis and treatment.

Between 1970 and 2010, 329 cases of thyroid hemiagenesis have been reported. Left lobe agenesia was more frequent with female’s predominance [19]. In humans the thyroid rudiment during thyroid development starts to acquire a bilobed structure at the end of the second month [20]. There are several known genes that control development and embryogenesis of thyroid gland but their role was not proven in hemiagenesis. In 1984 the case of thyroid hemiagenesis in two sisters was published [21]. Thyroid hemiagenesis could be found in some families suggesting genetic cause [22]. Certain familial hemiagenesis are caused by the transcriptional mutations of factors involved in embryogenesis.such as PAX8, TTF1,FOXE1, NKX2-5) [23]. In fact, only minority of cases of congenital hypothyroidism could be explained with such changes and it predominantly concerns cases of thyroid ectopy and agenesis, while in vast majority of patients with hemiagenesis the genetic background is unknown. GCMB gene is important for normal synthesis of parathyroid hormone in humans and could be involved in parathyroid adenoma genesis [24].

## Conclusion

Until now there was no case of thyroid hemiagenesis together with parathyroid adenoma and hyperplasia described in the literature. The case description proves that in a patient with thyroid hemiagenesis, despite unilaterally abnormal development of the thyroid gland, the parathyroid glands are present on the side of agenesis. The connection between parathyroid hyperplasia and adenoma and genetic triggers in their development needs to be clarified. The destiny of parafollicular C cells on the side of hemiagenesis is still unknown.

### Consent

Written informed consent was obtained from the patient for publication of this Case report and any accompanying images. A copy of the written consent is available for review by the Series Editor of this journal.

## Competing interests

The autors declare that they have no competing interests.

## Authors’ contributions

MO designed the manuscript, interpreted data and revised the manuscript. YI revised the manuscript and pointed out certain genetic links. MB and ID collected data, revised the manuscript. RD made diagnosis, operated the patient, followed up the patient, interpreted data and designed the manuscript. ZR discussed molecular basis of this unusual case and helped in revision and interpretation of data. GP made pathological diagnosis and photos of histopathological slides. All authors read and approved the final manuscript.

## Pre-publication history

The pre-publication history for this paper can be accessed here:

http://www.biomedcentral.com/1472-6823/12/29/prepub
